# Assessing the impact of public transfer payments on the vulnerability of rural households to healthcare poverty in China

**DOI:** 10.1186/s12913-022-07604-3

**Published:** 2022-02-23

**Authors:** Yali Li, Lei Huang

**Affiliations:** 1 School of Business, Jiangxi University of Science and Technology, Nanchang, 330013 China; 2 North Carolina State University, P. O. Box 8005, Raleigh, NC 27695-8005 USA; 3 School of Accounting, Jiangxi University of Finance and Economics, Nanchang, 330013 China

**Keywords:** Andersen model, Vulnerability to poverty, Multidimensional poverty, Multivariate logistic regression analysis, Public transfer payment, Chinese rural households

## Abstract

**Background:**

China completed the task of eliminating absolute poverty, following the 18th National Congress. However, after 2020, rural poverty in China has entered a new stage that is characterised by transformational secondary poverty and relative poverty; thus, the poverty vulnerable group is the new target group. Public transfer payments play a vital role in reducing the vulnerability of rural households to healthcare poverty. Assessing the effectiveness of public transfer payments in rural households can improve the vulnerability of rural households to healthcare poverty.

**Methods:**

In total, 5754 rural households were included each year, which accounted for a total of 16,722 rural households during the three-year study period. The multidimensional poverty and the vulnerability to healthcare poverty of rural households were assessed and compared. Two series of multivariate logistic regression models were further used to assess the effects of public transfer payments on improving the vulnerability of rural households to healthcare poverty.

**Results:**

When the poverty line was set at $1.90 and $3.20, rural households in all the three study years exhibited a higher vulnerability to healthcare poverty than the actual incidence of multidimensional poverty in healthcare, and the Eastern regions exhibited higher vulnerability to poverty than the Western regions of China. The series of multivariate logistic models employed to evaluate the effects of public transfer payments on the rural households’ vulnerability to healthcare poverty indicated that considering the differences in rural households’ demands for healthcare is vital for the government to fulfill the effects of public transfer payments. When income elasticity indicators for health care needs were included, the effect of public transfer payments on improving the vulnerability of rural households changed from less significant in 2014 and 2016. In 2018, however, the effect of public transfers on improving the vulnerability of rural households has increased compared to the non-inclusion elasticity.

**Conclusions:**

The imbalance of development between urban and rural areas in China is increasing, and rural households with heavy economic burdens are facing the risk of low healthcare services. Our findings highlight the importance of government departments in improving public transfer payments to reduce rural households’ vulnerability to healthcare poverty.

## Background

Poverty alleviation is a globally relevant topic that is crucial for achieving sustainable development goals. Poverty has been seriously threatening Chinese society. Eight years after the 18th National Congress (by the end of 2020), China completed the arduous task of eliminating absolute poverty in rural areas. According to official statistics, 98.99 million rural poor people were lifted out of poverty under current standards, 832 counties were removed from the list of poor counties, and regional overall poverty was resolved, thereby eliminating absolute poverty [1]. However, after 2020, rural poverty in China has entered a new stage that is characterized by transformational secondary poverty and relative poverty, which can markedly increase the percentages of the transformational poverty group and the potential poverty group [2]. With the complexity and diversity of human development, people’s understanding of poverty has been enriched. The governance of poverty has gradually developed from the initial solution to absolute poverty to influence individuals’ behavioral abilities under the perspective of multidimensional poverty [3]. When people could overcome the minimum threshold of basic security, they may suffer from deprivation in other key areas, which can hinder individual development, leading to vulnerability to poverty [4]. To effectively alleviate the vulnerability to poverty, several factors should be considered, in addition to exploring the mechanisms of poverty alleviation. Owing to the intergovernmental fiscal reform in 1994, the mechanisms of poverty alleviation played a non-negligible role in the management of local public finance. To overcome the deficit in fiscal spending on public services, local governments have had to resort to central government transfers and debt financing. Because the 1994 Budget Law prohibits local governments from borrowing through the budget, they have no choice but to rely on public transfers from the central government. Transfer payments can improve the financial situation of local governments by filling the fiscal deficit in public services [5] and regulating the public service provision behavior of local governments through incentives and accountability mechanisms [6].

In the modern era of high-stress economic development, workers may suffer from chronic diseases, such as bronchitis, and suboptimal physique owing to exertion due to long working hours and lack of adequate rest; these rural households are prone to become potentially vulnerable to healthcare for poor families. Simultaneously, the imbalance caused by the supply and demand of healthcare services and healthcare resources in many areas poses a health risk to the elderly population in rural households [7]. The impact of healthcare risk factors on poverty vulnerability includes both the micro and macro dimensions. At the micro-level, health risk shocks usually refer to the loss of family benefits caused by chronic diseases in elderly people or long-term working groups in the family over a specific period [8]. At the macro level, these factors refer to the risk impact of regional or global epidemic outbreaks, for example, the health impact on rural households under the current global corona-virus disease-2019 (COVID-19) outbreak [9]. These healthcare risk factors tend to contribute to the poverty vulnerability of rural households to healthcare. China has made remarkable achievements in eradicating absolute poverty, although much work is still needed for achieving relative poverty eradication goals, as evidenced by the fact that the elderly and long-term manual workers may be more vulnerable to potential healthcare vulnerability and that the reduction of public transfer assistance between families may increase the rural households’ vulnerability to healthcare poverty. Due to differences in the degree of demand for healthcare services in rural households, the effects of public transfer payments should be further assessed. Therefore, in this study, an effective analysis of the role and impact of public transfer payments on improving the rural households’ vulnerability to healthcare poverty was conducted, which included the income elasticity analysis of rural households’ demand for healthcare. The study may provide a scientific basis and guidance for China and other countries to effectively prevent and cope with rural households falling back into poverty.

The present study assessed a more forward-looking measure of welfare (vulnerability to poverty), as well as examined the relationship between income elasticity in healthcare needs and public transfer payments and the impact of this relationship on improving the vulnerability of rural Chinese households to healthcare poverty. Specifically, the study tested two empirical stages; first, the study used a methodology of vulnerability to quantify and assess the vulnerability of healthcare poverty, as household income may fall below the pre-specified poverty line in the future, and to explore the effect of public transfer payments on the poverty level of healthcare in rural China. Second, considering that the implementation of public mechanisms can have an impact on the well-being and subjective needs of policy beneficiaries. we measured the income elasticity of rural households’ demand for healthcare and used a multi-variable logistic regression model to evaluate and compare the marginal utility of the government’s fiscal transfer payment to the rural households’ healthcare poverty vulnerability before and after the inclusion of income elasticity of healthcare demands. The study provides evidence to Chinese public service decision-makers regarding whether the vulnerability of healthcare poverty in rural households is related to different levels of demand for healthcare services, thereby providing an in-depth understanding of the means of optimizing the allocation of healthcare resources for rural households by public transfer payments and proposing approaches to decrease vulnerability towards poverty.

### Literature review

With the complexity and diversity of human development, the understanding of poverty tends to be diversified. Studies have pointed out that some limitations exist in identifying poverty in monetary terms alone, and real poverty should be a denial of people’s current viability; people crossing the minimum threshold for basic economic security are eventually trapped in other key areas of individual development, including poverty vulnerability, resulting from the shortcomings of public service policies for sectors such healthcare [10]. ʻTransforming our World: The 2030 Agenda for Sustainable Developmentʼ was adopted by the United Nations [11]. In this agenda, the goal of “non-poverty”refers to addressing social, economic, and environmental poverty-associated factors in an integrated manner by measuring global multidimensional poverty indicators. The multidimensional poverty index (MPI) aggregates information on deprivation into 10 indicators to create a standard poverty assessment system [12]. With the COVID-19 pandemic and the associated economic burden, several scholars have revised the definitions of five indicators to better align with the sustainable development goals (SDGs) [13, 14]. From a multidimensional perspective, these poverty indicators can be divided into three dimensions: health, education, and standard of living, indicating that multidimensional poverty levels can be measured from these three dimensions [15]. The study of effective alleviation of multidimensional poverty must be implemented with appropriate governments support, which can play a crucial role in alleviating poverty [16]. Some scholars believe that public transfer payment can alleviate the effect of income poverty and income redistribution to the poor and extremely poor in general to a certain extent [3, 17, 18]. Moreover, some studies have explored its efficiency, and some researchers believe that by targeting fiscal transfer payments, poverty can be efficiently alleviated [19–23]. However, fiscal transfer payments can often lead to reverse incentives in the failure of poverty targets or misery in the implementation of public policies, leading to inefficiencies in efforts to alleviate poverty. Grosh conducted public transfer policy targeted efficiency research based on the anti-poverty status of 48 counties and found that of the 122 transfer payment projects, 25% of the transfer payment income is not accurately transferred to the poor, indicating considerable inefficiency [24]. Dabalen et al. evaluated the effects of the Albania’s anti-poverty public transfer policy in 1993 and used the propensity score matching (PSM) method to assess the welfare status of beneficiaries of continuous acceptance of transfer income [25]. Bargain et al. analyzed the effects of poverty alleviation in Finland by using repeated cross-sectional data from 1996 to 2003 and found that the use of the wrong measurement tools has a direct impact on the welfare eligibility and efficiency of the targets being assisted [26].

Multidimensional poverty analysis allows public sector decision-makers to assess the impact of a given plan or policy. However, according to studies, there are some reasons to make policies and approaches to be relatively unreliable and to consider their possible future impacts or outcomes [27, 28]. Anti-poverty policies have markedly attracted scholars’ attention in developing countries; thus, researchers believe that the key to policy success lies in accurately measuring the vulnerability to poverty [29, 30]. Vulnerability is the likelihood of an individual to have a level of welfare below some norm or benchmark at a given time in the future. Chaudhuri proposed that the concepts of vulnerability and poverty (which are also multidimensional) are linked but not identical; the author defined vulnerability as an ex-ante (forward-looking) concept and not as an ex-post concept [31]. In summary, three theoretical methods for measuring the vulnerability of poverty have emerged in the literature: Vulnerability as Low Expected Utility (VEU), Vulnerability as Expected Poverty (VEP), and Vulnerability as Uninsured Exposure to Risk (VER). The VEU’s measurement method was proposed by Ligon & Schechter [32]. This method measures the loss of well-being based on changes in utility and the vulnerability to poverty by the difference between the established level of equivalent consumption and the utility of consumption expectations. Later, the author selected data from the Bulgaria’s 1994 survey panel and used food expenditure as a measure of consumption levels to control individual heterogeneous characteristics, such as household income status, employment status, and the number of people receiving pensions, over time. The results showed that 55% of poverty vulnerability causes in the country were from low income, 13% causes were from synergy risk, 0.7% causes were from heterogeneous risk, and the remaining percentages of poverty vulnerability were caused by unexplained risks [32]. Thus, poverty plays a comparable role in reducing welfare levels. Poverty is an integral component of poverty vulnerability, accounting for more than a half of the overall vulnerability to poverty. Among the various types of risks that contribute to the vulnerability of family’s poverty, unexplained risks are the most important sources of vulnerability; therefore, estimating the breakdown of heterogeneous risks is necessary. A study by Klasen & Waibel in South-East Asia found that poverty vulnerability is caused by several factors, and more than a half of poverty vulnerability comes from low incomes, whereas the rest of the vulnerability arises from risks; additionally, the risks that are difficult to predict in the future are the most important sources of poverty vulnerability. Thus, an assessment of the risks to future survival and development of families is necessary [29].. Grech et al.’s research on European workers derived a similar conclusion [33]. Wald stated that higher-frequency data should be used when considering the impact of risk [30]. Other scholars have found that the most useful data for chronic poverty are the long-term data, such as long-term time-series data or panel data on household consumption [29, 32]. The measurement method of VEP was proposed by Chaudhuri, et al., Pritchett, et al., Hoddinott and Quisumbing [31, 34–36]. Subsequently, significant improvements in the method were made by Klasen & Waibel [29]. Pritchett et al. measured the vulnerability of poor areas in Indonesia. The authors defined poverty vulnerability as the likelihood of a family falling into poverty in approaching months [34]. The study defined the probability of future poverty to be higher than 50% as being vulnerable to poverty; thus, the authors set a threshold of 0.5. Moreover, the study analysed the regression data from Indonesia’s two-phase panel and found that the proportion of the total population at the risk of poverty vulnerability was much higher than that of the total poor population, with 10 to 30% of the population at a high risk of falling into poverty. In addition, poverty vulnerability may vary due to differences in sexes, education levels, regions, land ownership levels, sectors of the head of household, and other sectors [37]. However, this study on “poverty vulnerability group differences” could only qualitatively assess whether the households are poor and vulnerable, separating the more vulnerable families from the total population without quantitatively studying the vulnerability of these families towards poverty. Thus, the study could only reflect the total number of vulnerable families and not the depth of vulnerability to poverty in each family [38]. Given the revision of the VEP measurement method, Celidoni et al. pointed out that by assuming the risk of particularity to be independently distributed at different times and considering the presence of a common functional relationship between its degree of change and observable characteristics, the fixed effect of the family can be obtained based on the three-stage Feasible Generalised Least Square method [39]. Furthermore, Dutta et al. proposed a composite measurement method that considers the level of the poverty line and the current standard of living to set a baseline to measure poverty vulnerability [39]. The basic difference between the VEP and VEU methods is that the VEU method uses the change of utility to reflect the loss of the welfare level and infer poverty vulnerability, whereas the VEP method uses the estimate of income and consumption functions to infer poverty vulnerability. The VER measurement method was proposed by Dercon & Krishnan, who derived cross-sectional poverty statistics by using three consecutive semi-annual data from rural households in Ethiopia, which greatly underestimated the poverty vulnerability of households [40]. The VER method does not directly measure vulnerability but represents the degree of vulnerability by a sensitivity factor, and it does not separate the impact of risk on households from the magnitude of household response [30, 41].

The public transfer is also crucial for reducing poverty vulnerability in public services, such as healthcare, education, and social security. Imbalances are the main cause of poverty in developing countries. The pattern of contemporary economic development often results in a ʻransfer. outcome, and the tendency of public service resource input is positively correlated with the direction of economic resources. Consequently, families in marginal areas are more vulnerable to poverty in public services [42]. Specifically, in the field of healthcare public services, the general problem in developing countries is that the rural populations and families are much more vulnerable to healthcare-related poverty than the urban area populations due to deviations from the central radiation zone of the economy [43–45]. Therefore, public transfer payments from developed cities to backward rural areas were considered effective in reducing the vulnerability of healthcare-related poverty [46]. Although the aforementioned studies confirm the effectiveness of public transfer payments in improving vulnerability to poverty, concentrating on the efficiency of merit transfer payments in targeting beneficiaries is essential for reducing poverty vulnerability. The degree of demand for healthcare public services varies among the beneficiaries of different public mechanisms, and the implementation of early intervention on poverty alleviations is not possible without forward-looking projections of different levels of demand for beneficiaries of public services. Thus, the probability that these beneficiaries having a high level of demand for healthcare and undersupplied with public services will fall into poverty remains high [47]. However, the effect of public political transfer payments can be effectively enhanced by examining the welfare status of policy beneficiaries and different levels of demand for healthcare services [48]. Theoretically, different needs for improving poverty vulnerability are closely correlated to the demand income elasticity. Therefore, the scientific evaluation and study of the poverty vulnerability of the beneficiaries of public transfer payment improvement policies from the perspective of income elasticity in demand for healthcare services hold great practical significance. A literature search indicated that the vulnerability effects of public transfer payments on poverty from the perspective of demand income elasticity have been scarcely investigated. Therefore, we aim to assess the effect of public transfer payments on improving poverty vulnerability in rural households in China by using the China’s micro-survey panel data for 2014, 2016, and 2018. Based on Hashimoto & Heath’s calculation method on income elasticity of public services [49], we aim to further measure the income elasticity of healthcare demand and incorporate them into the analysis of the effects of public transfer payments on poverty vulnerability.

## Methods

### Data source

The data were derived from the national survey of the China Family Panel Studies (CFPS). China Family Panel Studies (CFPS) is nationally representative in China and with a bi- annually longitudinal survey of Chinese communities, families, and individuals conducted in 2010 by the Institute of Social Science Survey (ISSS) of Peking University, China. CFPS is an ongoing annual longitudinal survey of Chinese communities, families, and individuals launched in 2010 (wave 1), followed up in 2012 (wave 2), and 2014 (wave 3), then in 2016(wave 4), and updated in 2018 (wave 5). The year of the study was 2014, 2016, and 2018 respectively. In this study, we selected the CFPS data for 2014, 2016, and 2018 respectively to assess the vulnerability of rural households to health care poverty and study the financial effects for the goal of reducing vulnerability. The CFPS survey of 2014, 2016, and 2018 respectively adopted a multi-stage probability sampling, with more than 1800 villages in 28 provinces of China as the primary sampling units, and recruited 13,996 households representing 95% of the national population, including 7252 urban households and 6744 rural households. Our sample was restricted to rural households which benefited from China’s current anti-poverty policy. It is worth noting 990 respondents with missing values of health care access were excluded. This resulted in a total of 5754 seniors.

### Main dependent variables

In the research process, the key-dependent variables are first constructed. It contains the Expected Poverty Vulnerability Index for Health Care for Rural Households in 2014, 2016, and 2018, collects variable information based on household income, health care poverty identification, head-of-household identification, etc. from the CFPS Family Economy Module and the Personal Module to estimate the vulnerability to expected poverty by the method of VEP. The basic formula for calculating vulnerability put forwarded by [31, 50] is as follows:

1 $$\hspace{0.25em}{\mathit{VUL}}_{\mathit{ht}}=P_r\left(C_{h,t+1}\leq poor)\right.$$

Specifically, they define welfare in terms of consumption so that vulnerability of household h at time t-*V*_*ht*_- is the probability that the household’s level of consumption at time t + 1 (*C*_*ht* + 1_) will be below the consumption poverty line, poor.

Consumption t + 1 can be expressed as observable variables (*X*_*h*_) and the functions of error terms containing shock factors (*e*_*h*_). The formula for consumption t + 1 is as follows:

2 $${C}_{h,t+1}=f\left({X}_h,{\alpha}_h,{e}_h\right)$$

The estimation strategy of [31] and the three-stage feasible generalized least-squares method of [51] are used in this paper. The first step is to estimate the consumption equation, i.e. the following formula exists:

3 $$Ln{C}_{h,t}={\alpha}_h{X}_{h,t}+{e}_h$$

Among them, *C*_*h*, *t*_ represents the consumption of individual h in the t period, *X*_*h*, *t*_ expresses some individual or family characteristic variables. In this paper, we mainly include the following variables, namely age, education level, gender, family size, etc. The predictive dependent variable $\hat{C}=$  *C*_*h*, *t*_ and residual term *σ*_*e*, *h*_ can be obtained by using formula (3).

The second step estimates the sum of the expected $\hat{E}$ and variance of ${\sigma}_{e,h}^2={X}_h\beta$ for the logarithm consumption expressed as the following:

4 $$\hat{E}=\left(\mathit{\ln}{C}_h|{X}_h\right)={X}_h\hat{\alpha}$$

5 $$\hat{V}\left(\mathit{\ln}{C}_h|{X}_h\right)={\sigma}_{e,h}^2={X}_h\hat{\beta}$$

The third step assumes that consumption obeys the normal distribution of the numbers, then, the vulnerability calculations can be reduced to the following:

6 $$\hat{\mathrm{VU}{\mathrm{L}}_{\mathrm{h}}}={\hat{\mathrm{P}}}_{\mathrm{r}}\left(\ln {\mathrm{C}}_{\mathrm{h}}\le \ln \mathrm{poor}\right)=\upvarphi \left\{\ln \mathrm{poor}-{\mathrm{X}}_{\mathrm{h}}\hat{\upalpha}/{\left({\mathrm{X}}_{\mathrm{h}}\hat{\upbeta}\right)}^{1/2}\right\}$$

### Main independent variable

We build the key independent variables. It is divided into two parts of variables. The first part is the government public transfer payment (GTP), from the CFPS family economic survey to collect whether the rural household received the public transfer payment and received the amount of public transfer payment. Of these, each survey year for each of the 3 years is recorded as 1 for households receiving public transfer payments, while other cases are marked as 0. The second part is the elasticity of demand income for health care (E_H) and the interaction between public transfer payment and that elasticity (GTP*E_H). To define the income elasticity of demand for health care. “Income Elasticity of Demand” refers to the extent to which changes in consumers’ income affect changes in demand for a commodity, or how changes in demand for a commodity react to changes in consumer income over some time, subject to the same conditions. “Income Elasticity of Demand on Public Goods,” can be expressed as the extent to which changes in consumer income in public services affect changes in demand for public goods over a while, subject to other conditions. This paper mainly studies the income elasticity (E_H) of rural households’ demand for health, and the demand for public services is the demand for public services that consumers are willing and able to pay, that is, the demand for available currency under the constraints of each household budget [49]. Thus, the money that rural households pay for healthy public services is the demand for health public services. According to the annual household health expenditure obtained from the family economy module of the CFPS database, the demand for the health of rural households is obtained, and the annual income of rural households is in the amount of income of consumers who consume such goods.

### Covariates

In collecting and using CFPS family-level data, information on property owners was asked from the 2010 household questionnaire that the head of the household was generally referred to as the name or first person to appear on the family property or land certificate. Since 2012, the screening of heads of the household has added a survey of financial managers in the household questionnaire, which found that the person most familiar with all family members and able to answer some of the family finances in the past year was also the head of the household. Because of this, this paper combines the family real estate owners and financial managers to answer the information, 5754 rural households for the identification of the head of household.

Developed in the United States in 1968 using healthcare behavioral models, The Andersen model has been widely used in international and U.S. health service research [52, 53]. It is used to guide systematic investigations into the factors that lead to the use of health services, including predisposing, enabling, and health-need factors. In understanding the utilization of health care in China, we must consider the actual situation of health care in rural Chinese households, based on the three major factors of health care service included in the Andersen healthcare behavioral model, and determine the control variables in this paper when studying the multidimensional poverty and vulnerability to poverty of health care.

Predisposing factors can be characteristics of the head of household including age (over 17 years of age and under 92 years of age), gender (male/female), years of education (under 1 year- illiterate/1 ~ 6 years-primary school level/6 ~ 9 years-junior school level/ 9 ~ 12 years-senior school level/12 ~ 16 years- undergraduate level/more than 16 years-graduate level) [52]. Besides, also include the number of family members and the square of the head of households’ age as the control variables of this study to ensure the stability of the regression results.

Enabling factors can be measured as tangible resources of obtaining health care services, such as household net income, access to health insurance (yes/no) [52]. Among them, the variable of household net income is the key variable applied to the calculation of income elasticity of rural households’ demand for health care. And the variable of access to health insurance is applied to the identification of health and medical multi-dimensional poverty in rural households.

Health-need characteristics can be assessed by chronic illness in the last 6 months (yes/no), bronchitis illness in the last 6 months (yes/no), asthma illness in the last 6 months (yes/no), hospitalized for the last 12 months (yes/no), health status by perceived evaluation (0 ~ 4 score-poor or fair/4 ~ 7good or excellent) [52]. These are all key variables in measuring whether rural households have multidimensional poverty in health and health care.

### Statistical models

#### Multidimensional poverty identification model for health care

Referring to the A-F method [54], the identification method consists of three steps. The deprivation critical vector of indicator “n” is determined at the first step, that is *Z*_*n*_ = (*Z*_1_, *Z*_2_, ⋯, *Z*_*j*_)^*t*^. It is assumed that ${\rho}_{mn}^t$ is an identification value for a single vector, for any matrix ${\mathrm{Y}}_{\mathrm{i}\times \mathrm{j}}^{\mathrm{T}}$. When ${y}_{mn}^t<{Z}_n$, it indicates that the rural family “m” is recognized as poverty during the t-period, and counted as ${\rho}_{mn}^t=1$, otherwise, counted as ${\rho}_{mn}^t=0$. The second step is to determine the indicator weight vector, that is, W_n_ = (w_1_,  w_2_, ⋯, w_j_). The W_n_ is the nth indicator weight, and meet the condition of the equation, that is $\sum_{\mathrm{n}=1}^{\mathrm{j}}{\mathrm{w}}_{\mathrm{j}}=1$. Then by constructing the equation of the weighted deprivation matrix C, that is, ${c}_m^t=\sum_{\mathrm{n}=1}^{\mathrm{j}}{\mathrm{w}}_{\mathrm{j}}{\rho}_{mn}^t$, and the family “m” was deprived of the score on all the indicators during the t-period. At the third step, the multidimensional poverty threshold vector “k” is set, and when the condition is reached, that is, ${c}_m^t\ge \mathrm{k}$, the family “m” is recognized as a multidimensional poor family during the t-period. The multidimensional dimension of poverty in health and health care selected in this study is obtained by referring to the Multidimensional Poverty Index (MPI) index system conducted by the Oxford Poverty & Human Development Initiative (OPHI) in the UK and based on the context of China’s actual development [55]. Therefore, based on the fact that Chinese rural households are deprived of health care and the availability of CFPS database data, the family members in the past 6 months whether there are chronic illness, bronchitis illness, asthma illness, hospitalization, and self-reported health status as a measure of rural family health deprivation indicators, and in the past year whether family members have participated in medical insurance as an indicator to measure rural family medical deprivation, to build in line with the current reality of China’s rural family health and medical multidimensional poverty dimension, indicators, deprivation threshold, and weight (see Table 1). In this paper, based on [15]‘s weight setting method, two dimensions are assigned equal weights, reflecting a normative judgment of equal importance to capture multidimensional poverty. We also set the multi-dimensional poverty rate according to the official poverty threshold (−deprivation in three or more indicators or k =30%). When the proportion of the six indicators values exceeds 30%, it is considered that the household is in a multi-dimensional poverty state (m = 1), and vice versa (m = 0).

**Table 1:** Multidimensional poverty indicator system for health care

Dimensions	Indicators	Threshold of deprivation	Weight
Enabling	health insurance	Access to the health insurance(Yes = 0, No = 1)	1/6
Health need	Chronic illness	Whether or not chronic illness has been in the last six months(Yes = 1, No = 0)	1/6
	Bronchitis illness	Whether or not bronchitis illness has been in the last six months(Yes = 1, No = 0)	1/6
	Asthma illness	Whether or not asthma illness has been in the last six months(Yes = 1, No = 0)	1/6
	Hospitalization	Whether or not entering the hospital in the past 12 months (Yes = 1, No = 0)	1/6
	Health status	The health status by self-reported (range from 0 ~ 7score, below 4 as poor and over 4 as good health level)	1/6

Source: CFPS 2014,2016 and 2018 based on A-F method and Andersen model [52, 54, 56]

### Health care vulnerability as expected poverty model (VEP)

In this study, we used the method of correcting selective and endogenous bias to assess the impact of government public transfer payments on the health care poverty of rural households in China. Based on the VEP method [31, 36, 50], we tried to provide examples where vulnerability is defined as the probability that a rural household will fall into health poverty in the future. The basic principle of VEP is the return of observable variables and impact factors to the per capita health care consumption of the family to obtain the expected health care consumption per capita of the family, and then assume that the expected health care consumption per capita of the family obeys the normal distribution of the number, thus obtaining the probability that the expected health care consumption per capita of the future family is lower than a certain value (usually the poverty line), that is, the probability that the family will fall into poverty in the future. By using Chaudhuri’s estimation strategy [31] and the three stages of the method proposed by Amemiya [51], it can be feasible generalized least squared (FGLS), the vulnerability of health poverty can be estimated through three stages. The first step is to estimate the equation of health care consumption and the equation of residuals. The following variables are adopted, including multi-dimensional poverty (yes = 1, no = 0), age, educational year, marital status (married = 1, unmarried = 0), family size, etc., and to control the fixed effect between regions, we also analyze the regional variables represented by dumb variables. The second step uses the fitted value obtained in the first step to build the weight for FGLS estimation. The third step is to select the poverty line, based on panel data for 2014, 2016, and 2018, we estimate the results of healthy poverty vulnerability based on the $1.9 and $3.2 the two standard poverty lines [57]. It is worth noting that in the estimates of VEP, we have adopted the poverty line of $1.90 and $3.20. And to calculate the robustness, this paper uses two criteria based on the calculated international poverty line, namely, US$1.9/day per capita, US$3.2/day per capita, and obtains a new international poverty line measured in Chinese currency (about RMB 2900 and 5000), in combination with the exchange rate and the adjustment of urban and rural living cost according to the different regions in China provided by the database. Besides, it is also worth noting that in vulnerability studies, the threshold determination of vulnerability is subjective and arbitrary, so we set a threshold of vulnerability line based on Pritchett’s research result [34]. It is set the predicted probability that per capita health care for individual households is below 50% of the poverty line as the expected vulnerability for health care. if the predicted probability of per capita log health of individual households is below 50% of the poverty line as the threshold for vulnerability,

### The multivariate logistic regression model

Multivariable logistic regression analysis performed with STATA 15.0 was employed to examine government public transfer payment associated with the marginal effect on the vulnerability of health care poverty in rural households in China. Two series of logistic regression analyses were undertaken. In the first series, based on the factor variables as a di-factor, the factor is the vulnerability of health care expectation poverty (poverty vulnerability = 1, non-poverty vulnerability = 0). The multi-logistic regression model is selected to study the effect of series one: public transfer payment on the poverty vulnerability of health care in rural China under the condition of controlling the specific characteristics of the head of household. In the second series, the weighted value of household income elasticity to health care demand is measured as the key argument of series two, and the interaction between income elasticity of health care demand and government public transfer payment is constructed. It is incorporated into the logical model of phrase one to examine whether the improved marginal effect of the public transfer payment on the vulnerability of health care poverty in rural households in China is related to the degree of subjective demand for health care in the families, and to make empirical analysis.

## Results

### The measurement analysis of the vulnerability of health care poverty

As can be seen from Table 2, the incidence of multidimensional poverty, as measured by health and medical deprivation *R*^*c*^, is on the rise (k = 30%, from 38.62 to 44.27%, and k = 40%, from 9.85 to 17.60%), and it is clear that, with the development of the economy and society, the main contributors to poverty have gradually shifted from the demand for basic living to the level of different needs for health and medical care, with the development of the economy and society and the continuous improvement of the quality of family life and living standards. Health and medical care have an important impact on the incidence of multidimensional poverty throughout the study period, and how to meet the different needs of rural households for health and medical care on a larger scale has become a key issue for China to consolidate the achievements of poverty eradication and promote the vigorous development of rural areas. Since the values of the poverty vulnerability indicators for 2014, 2016, and 2018 are influenced by factors other than age, gender, marital status, and length of education in addition to multidimensional poverty, the poverty rate is not much different from the estimated poverty vulnerability values of 30% or 40%. Therefore, we have selected a poverty rate of 30%to measure poverty vulnerability. And the vulnerability of China to health care poverty has been declining year by year in all three study years., and the gap between poverty vulnerability and the incidence of multidimensional poverty (k = 30%) is narrowing year by year, especially in 2018, the vulnerability to health care is 50.78 and 57.13%, respectively, a difference of 6.51 and 12.86%.

**Table 2:** Compare the vulnerability and incidence of health care poverty in 2014, 2016, and 2018

Main statistics	The Year 2014		The Year of 2016		The Year of 2018	
Multidimensional poverty threshold (*k*%)	*k* = 30%	*k* = 40%	*k* = 30%	*k* = 40%	*k* = 30%	*k* = 40%
Incidence of multidimensional poverty in health care (*R*^*c*^)	38.62%	9.85%	40.32%	11.66%	44.27%	17.60%
Health care experts poverty vulnerability (Poverty line = $1.9, *𝑘=30%*)	93.08%		92.09%		50.78%	
Health care experts poverty vulnerability (Poverty line = $3.2, *𝑘=30%*)	96.28%		96.02%		57.13%	

Data source: Authors’ calculation using CFPS 2014, 2016, and 2018 [56].

Note: k = 30 % , k = 40% represent the threshold for setting multidimensional poverty is 30 and 40%, respectively. The incidence of multidimensional poverty R^c^ indicates the proportion of poor households $\left({f}_{\mathrm{m}}^{\mathrm{c}}\right)$ in the year understudy to the total sample size of households (i), and the formula is: ${R}^c=\frac{\sum_{m=1}^i{\delta}_m\left(k,\gamma \right)}{i}=\frac{f_m^c}{i}$

**Fig. 1:**
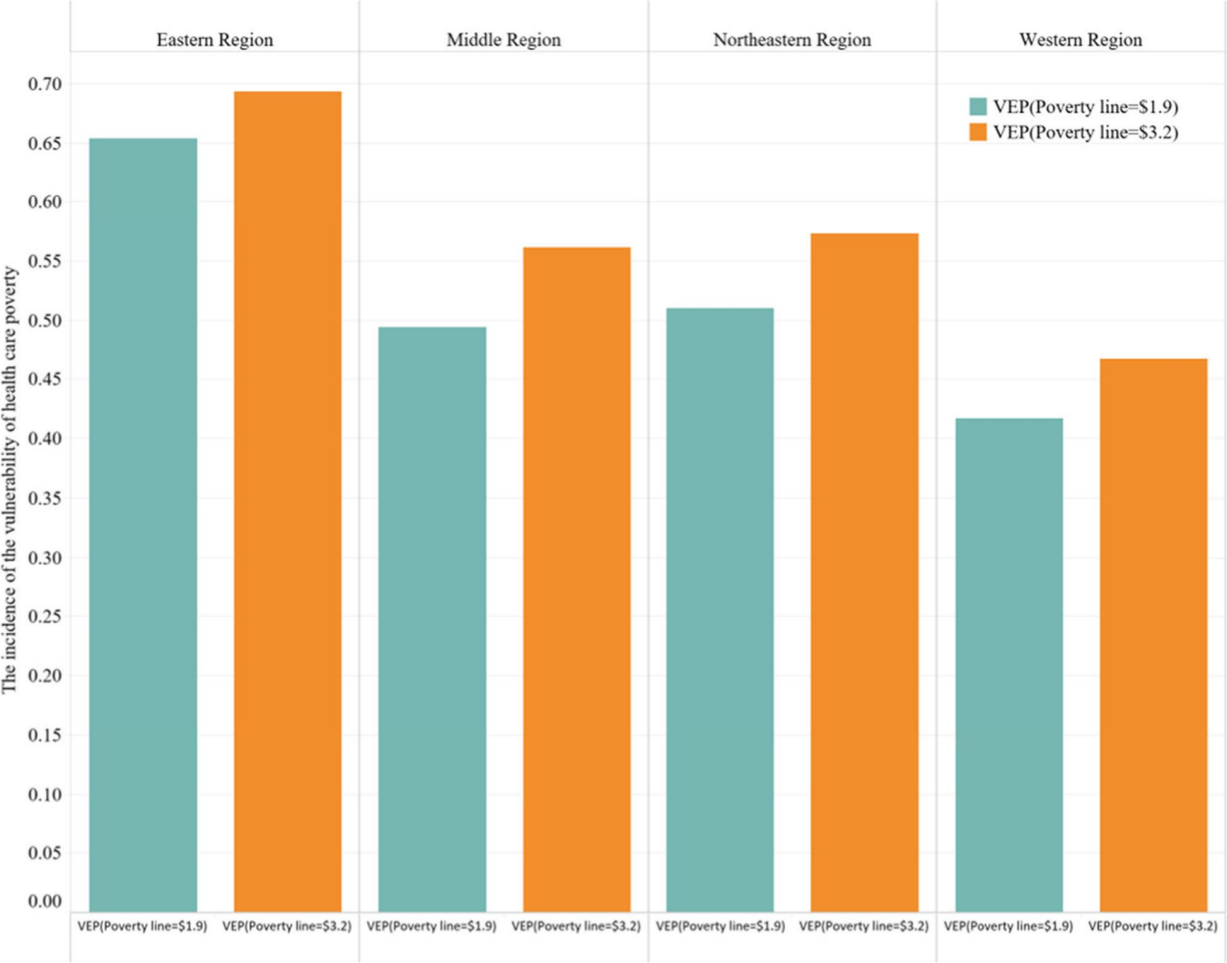
The incidence of the vulnerability of health care poverty is shown in the eastern, middle, western, northeastern, and western regions of China (there are 28 provinces and cities in total due to the difficulty of data acquisition in other provinces which divides all provinces in China into four regions: east, middle, northeast, and west) [52]

Regional poverty disparities are common in many developing economies especially pronounced in some such as China. In China, for example, counties classified as national or provincially poor (assessed according to poverty level) selectively receive additional government financial support. It is very noteworthy that in some provinces or cities with high rates of poverty, the incidence of poverty is much higher than the national poverty standard. However, the estimated incidence of poverty vulnerability in these areas with high incidences of poverty may be well below the national poverty vulnerability criteria. Table 3 reflects the incidence of poverty vulnerability in China’s rural geography at the $1.9 and $3.2 vulnerability criteria based on 2018 CFPS’s data. As can be seen from Table 3, China is classified as a poor Yunnan, Tibet, and other regions, and in this study to calculate the average incidence of multidimensional poverty in health care, Yunnan and Tibet have a poverty incidence rate of well above 60%, indicating a high incidence of poverty. However, the expected poverty vulnerability assessed under the $1.9 and $3.2 vulnerability criteria in the two regions were divided into low vulnerabilities to health care. Similarly, cities with low incidences of poverty, such as Beijing and Shanghai, are classified as highly poor vulnerabilities to health care, as assessed in the $1.9 and $3.2 vulnerability criteria. To further analyze and compare the vulnerability of rural Chinese families to health care in different regions, Based on the importance of spatial geographical distribution, we divide the data from 28 provinces and cities into the eastern, central, western and northeastern regions of China to examine the vulnerability of rural family health care poverty in four regions of China in 2018 (see Fig. 1). We find that when the poverty line standard is $1.90 or $3.2, the expected measure of rural family health care poverty vulnerability is the lowest in western China, followed by the central region, and the highest poverty vulnerability index is China’s highest level of economic development and low-income poverty in the eastern regionrural households.

**Table 3:** Classification of the high, medium, and low vulnerability provinces or cities in 2018 survey wave

Class	Range of vulnerability (Average Value)	Provinces or Cites of VEP (Poverty line = $1.9)	Provinces or Cites of VEP (Poverty line = $3.2)
High vulnerability	60% ~ 70%	Beijing, Shanghai, Zhejiang, Jiangsu, Hainan, Shangxi	Beijing, Shanghai, Zhejiang, Guangdong, Jiangsu, Hainan, Heilongjiang, Chongqing, Hunan, Yunnan, Shandong, Hebei, Shangxi
Moderate vulnerability	50% ~ 60%	Guangdong, Heilongjiang, Chongqing, Hunan, Yunnan, Fujian, Shandong, Hebei, Anhui, Shanxi, Liaoning	Fujian, Henan, Hubei, Jiangxi, Anhui, Guizhou, Shanxi, Sichuan, Gansu, Liaoning
Low vulnerability	Below 50%	Henan, Tianjin, Hubei, Jiangxi, Guangxi, Guizhou, Sichuan, Xinjiang, Tibet, Gansu, Jilin	Tianjin, Guangxi, Xinjiang, Tibet, Jilin

Source: VEP estimation method using CFPS 2018 [31, 34–36, 56]

### Multivariate logistic regression

Table 4 lists the descriptive statistics of the effect of public transfer payments on vulnerability to health care poverty in Chinese rural households.

**Table 4:** Descriptive statistics for variables

Variables	Year	VEP_1 (Poverty line = $1.9)	VEP_2 (poverty line = $3.2)	(GTP) (Received = 1, Non-received = 0)	Age	Age^**2**^	Gender (Male = 1, Female = 0)	Educational Year (Y_E)	Family Size (F_ S)
Statistics									
Minimum	2014	0	0	0	17	2.89	0	0	1
	2016	0	0	0	17	2.89	0	0	1
	2018	0	0	0	17	2.89	0	0	1
Maximum	2014	1	1	1	92	85	1	19	21
	2016	1	1	1	92	85	1	19	21
	2018	1	1	1	92	84.64	1	19	21
Mean	2014	0.93	0.96	0.72	49.92	26.77	0.76	5.79	3.92
	2016	0.92	0.96	0.59	49.92	26.77	0.76	5.79	3.92
	2018	0.51	0.57	0.6	49.92	26.77	0.76	5.79	3.92
Std. Deviation	2014	0.25	0.18	0.45	13.58	13.6	0.42	4.83	2.01
	2016	0.27	0.19	0.49	13.58	13.66	0.42	4.83	2.01
	2018	0.5	0.49	0.48	13.58	13.66	0.42	4.83	2.01
Number of Sampling	2014	5754							
	2016								
	2018								

Source: CFPS 2014, 2016 and 2018 [56]

### Series one model:multi-variables logistic model effect analysis

Series one is a study of the marginal effect of government public transfer payment on the vulnerability of health and hygiene poverty in rural households. Based on the particularity of the Logistic model, the contribution rate of fiscal transfer payment to vulnerable poverty is understood more directly, so this paper translates the regression result into marginal effect. As can be seen from the marginal effects regression results in Table 5, when the poverty line was set at $1.90 and $3.20, government public transfers in 2014 and 2016 had almost no effect on health care vulnerability in rural areas. And in 2018 the government’s public transfer payments have significantly improved the vulnerability of rural households to health care poverty. This may be because in response to China’s actual development, the overall level of rural economic development in China was low in 2014 and 2016, with local governments paying far more to cities than in rural areas due to regional political and economic tournaments. As a result, the scope of the Chinese government’s subsidies to rural households is mainly concentrated in agricultural subsidies and limited strength, in these two study years, the proportion of rural households in line with health care vulnerability to poverty than the proportion of families receiving public transfer payments. Besides, local governments fail to take into account the level of demand for health care in rural households, resulting in a serious underpayment of transfers in this area, which is why government public transfer payments, whether set at $1.9 or $3.2 in 2014 and in 2016, have little impact on health care vulnerability in rural areas. In 2018, the government’s fiscal transfer payments contributed to a decline in rural household health care vulnerability to poverty, while when the poverty line was set at $1.9 and $3.2, the vulnerability of rural households that received public transfer payments in 2018 fell by 3.4 and 6.04%, respectively. This may be because the proportion of poor households that meet the vulnerability of health care will be smaller in 2018 than the proportion receiving public transfer payments. From the control variables, it can be found from the control variables that, during 2014, 2016, and 2018 year surveys, empirical results found that female-headed households with younger age and a higher level of education were more responsive to reducing the vulnerability of health care through public transfer payments. And vulnerability to poverty was found to be higher among small-scale rural households than in large-scale rural households during the 2014 and 2016 year surveys, but this result was not valid in 2018, and in 2018 large-scale rural households were found to be more responsive to public transfer payments reducing the vulnerability of health care. This may be because with the economic development level of various regions of China has increased significantly recently, the larger the number of families, the greater their ability to jointly fight the risks of future health care, and also related to the three-year study year of health care vulnerable poor families accounted for a different proportion of the overall rural household sample.

**Table 5:** Logistic regression results on the vulnerability effect of fiscal transfer payments on rural family health care poverty

Variables	VEP_1 (Poverty line = $1.9)			VEP_2 (poverty line = $3.2)		
	2014	2016	2018	2014	2016	2018
Statisitics	Coefficient	Coefficient	Coefficient	Coefficient	Coefficient	Coefficient
	*p* -value	*p* -value *|*	*p* -value	*p* -value	*p* -value	*p* -value
GTP	−0.0105 (0.139)	−0.0015 (0.831)	−0.0340^***^ (0.010)	− 0.0031 (0.157)	−0.0048 (0.354)	− 0.0604^***^ (0.000)
Age	0.0014 (0.257)	0.0019 (0.151)	0.0044^*^ (0.099)	0.0002 (0.788)	0.0006 (0.560)	0.0002 (0.925)
Age^2^	0.0016 (0.187)	0.0024^*^ (0.066)	0.0016^**^ (0.045)	0.0003 (0.739)	0.0002 (0.762)	0.0044^*^ (0.087)
Gender	0.0221^***^ (0.004)	0.0232^***^ (0.005)	−0.1028^***^ (0.000)	0.0149^***^ (0.008)	0.0178^***^ (0.002)	0.0799^***^ (0.000)
Y_E	−0.0009 (0.181)	−0.0005 (0.502)	− 0.0113^***^ (0.000)	−0.00003 (0.949)	− 0.0014^**^ (0.016)	−0.0122^***^ (0.000)
F_S	0.021^***^ (0.000)	0.0131^***^ (0.000)	−0.0152^***^ (0.000)	0.0153^***^ (0.000)	0.0131 ^***^ (0.000)	−0.0457^***^ (0.000)
Log likelihood	− 1352.12	− 1514.91	− 3909.70	− 843.12	− 891.95	− 3688.69
LR Statistics	189.85	152.19	155.91	142.54	141.43	482.09
Number of Sampling	5754	5754	5754	5754	5754	5754

Source: CFPS 2014, 2016 and 2018 [56]

Note: *, **, *** represented statistic indicators significantly at 10, 5, and 1%, respectively

We can see that during the three expeditions in 2016, 2014, and 2018, the targeting mechanism for poverty demand for health care vulnerability due to public transfer payments was inadequate. Government public transfer payments have contributed only to the decline in poverty in rural family health care vulnerability. This may be because members of the family have different levels of demand for health care, and government transfer payments do not change accordingly to the subjective needs of the family, and therefore may lead to transfer payments that are less effective in alleviating the vulnerability of health care poverty. To further measure the effectiveness of public transfer payment on rural family health care vulnerability to poverty, the income elasticity of rural households’ health care needs is analyzed in the empirical evidence of series two and incorporated into the same model.

### Series two model:multi-variables logistic model effect analysis

At the first step, estimate the income elasticity of demand for public goods. To study the demand relationship between rural household income and health public service, this paper constructs a double-to-scale model, taking the natural icing of rural household income as the key argument, and the natural icing on the demand for health care as the dependent variable, establishing the general regression equation and obtaining the argument coefficient, to measure the income elasticity of the family’s demand for health care. In the second step, do the empirical test of interaction effects. Before analyzing the interaction between the two variables of income elasticity and public transfer payment of health care demand on the poverty effect of rural family health care, it is necessary to line-check whether the variables composed of interactive items have an interaction effect and to test whether the interactions in the two models of $1.9 and $3.20 in poor counties are significantly combined, i.e. to test the original hypothesis that the coefficient of the interaction items is all 0. It can be seen that the interaction item is the interaction effect between the family’s income elasticity and the public transfer payment for health care needs (GTP*E_H).

As can be seen from the test results of Table 6, in the models of the poverty line of $1.9 and $3.2, the Prob. value of the interactive item introduced by GTP*E_H is less than chi2, and both are significantly significant at the confidence level of 0.1, rejecting the assumption that the coefficient of the interaction term is all 0. It is indicated that the joint significance test of this interaction item has passed [58]. As derived from Table 6, the Logistic model estimates that the marginal effect of fiscal transfer payments on rural household health vulnerability to poverty is effective.

**Table 6:** The interaction effect of the joint variable

Variables	GTP*E_H (VEP_1)	GTP*E_H (VEP_2)
Wald(Prob.)	0.0000^***^	0.0000^***^
Chi2 (1)	261.61	173.17

Source: CFPS 2014, 2016 and 2018 [56]

Note: *, **, *** represented statistic indicators significantly at 10, 5, and 1%, respectively

From the return results of Table 7, it can be seen that in 2014, 2016, and 2018 survey waves, there was a significant positive correlation between the income elasticity of rural households’ demand for health care and the health care vulnerability in rural family, indicating that the greater the elasticity of rural households’ demand for health care, which means that these families’ constant pursuit of health levels drives them to respond to future health and medical risks so that these families are more able to cope with negative impact risks and deal with risks, and the lower the probability of suffering from health care vulnerability to poverty in the future. Besides, after adding the income elasticity variable of rural households’ demand for health care in the three study years, the interaction between demand income elasticity and public transfer payment showed a significant positive relationship with the decline of rural family health care vulnerability. From the regression coefficient values, the marginal utility of the interactions in 2014, 2016, and 2018 to improve the vulnerability of rural household health care to poverty risk at $1.90 is 0.0258, 0.5279, and 0.1981 respectively, and the marginal utility of poverty risk to improving the vulnerability of rural family health care at $3.20 is 0.0124, 0.3205, and 0.2283 respectively. In 2014 and 2016, as households’ demand for health care increased, the effect of public transfer payments on improving their vulnerability changed significantly from the inability to incorporate demand income elasticity into the significant effect of inclusion in the elasticity to improve their vulnerability. In 2018, the effect of public transfers on improving their vulnerability will increase when they are not included in demand income elasticity. It means that the local government’s public transfer payment means to enhance the targeting mechanism of the poverty demand of health care vulnerability, the demand of rural households for health care is increasing year by year, and the proportion of its members in sub-health states and suffering from chronic diseases is increasing year by year, which means that the actual cost and opportunity cost of health care will continue to increase, and if more attention is paid to the degree of rural households’ demand for health care, it will help rural households to improve their ability to cope with the risk of health care. Therefore, fiscal transfer payments have significant positive effects on improving the vulnerability of health care to poverty.

**Table 7:** Marginal logistic regression results of the poverty effect of the fiscal transfer payment on rural family health care vulnerability

Variables	VEP_1 (Poverty line = $1.9)			VEP_2 (Poverty line = $3.2)		
	2014	2016	2018	2014	2016	2018
E_H	−0.0281^***^ (0.000)	− 0.0220^***^ (0.000)	− 0.0791^***^ (0.000)	−0.0174^***^ (0.000)	− 0.0512^***^ (0.000)	−0.0925^***^ (0.000)
GTP*E_H	−0.0258^***^ (0.000)	− 0.5279^***^ (0.000)	− 0.1981^***^ (0.000)	−0.0124^***^ (0.009)	− 0.3205^***^ (0.000)	−0.2283^***^ (0.000)
Age	0.0006 (0.602)	0.0009 (0.400)	0.0042^*^ (0.100)	0.0002 (0.770)	0.0007 (0.421)	0.0004(0.856)
Age^2^	0.0009 (0.437)	−0.0003 (0.746)	− 0.0014^*^ (0.588)	0.0001 (0.913)	0.0009 (0.251)	0.0052^**^ (0.043)
Gender	0.0217^***^ (0.003)	0.0075^*^ (0.070)	−0.1026^***^ (0.000)	0.0154^***^ (0.005)	0.0108^**^ (0.028)	−0.0822^***^ (0.000)
Y_E	- 0.0004 (0.511)	−0.0015^**^ (0.026)	−0.0096^***^ (0.000)	− 0.00036 (0.514)	−0.0005 (0.347)	0.0105^***^ (0.000)
F_S	0.0229^***^ (0.000)	0.0199^***^ (0.000)	−.0133^***^ (0.000)	0.0158^***^ (0.000)	0.0136^***^ (0.000)	−0.0440^***^ (0.000)
Loglikelihood	− 1288.93	− 1022.16	− 3793.30	− 802.56	− 626.31	− 3524.67
LR Statistics	316.23	1137.70	388.73	223.67	672.69	810.15
Number of Sampling	5754	5754	5754	5754	5754	5754

Source: CFPS 2014, 2016 and 2018 [56]

Note: *, **, *** represented statistic indicators significantly at 10, 5, and 1%, respectively

### Cross-validation regression

To ensure the robustness of the regression results, the paper replaces the poverty rate with the original k-30% to k-40% by transforming the interpreted variables and the replacement estimation method and uses the random effect Probit(robust) model to regress the vulnerability to poverty marginal effect of the two models VEP_1 and VEP_2. Table 8 reports the results of the robust test analysis, and the regression results show that the household head characteristic variables are controlled, and after adding the interaction effect between the family’s income elasticity to health care needs and the public transfer payment, except for the two control variables of age and age squared in 2014 and 2016, the other key variables are non-significant, namely, the income elasticity of household demand for health care, the interaction effect between family income elasticity of health care needs and public transfer payment, and the gender of the head of household. Education and family size are highly significant at the level of 10, 5, and 1%, respectively. And the coefficient symbol of the variable is consistent with the series Logistic models, and the models have passed the Wald test, which shows that the conclusion of introducing the income elasticity of family demand for health and analyzing the poverty effect of the public transfer payment on rural family health care vulnerability is stable and reliable.

**Table 8:** Marginal porbit(robust) regression results of the poverty effect of the fiscal transfer payment on rural family health care vulnerability

Variables	VEP_1 (Poverty line = $1.9)			VEP_2 (poverty line = $3.2)		
	2014	2016	2018	2014	2016	2018
E_H	−0.0302^***^ (0.000)	− 0.1371^***^ (0.000)	− 0.1510^***^ (0.000)	−0.0184^***^ (0.000)	− 0.0578^***^ (0.000)	−0.1502^***^ (0.000)
GTP*E_H	−0.0356^***^ (0.000)	−0.5946^***^ (0.000)	− 0.1991^***^ (0.000)	−0.0183^***^ (0.001)	− 0.3210 ^***^ (0.000)	− 0.2261^***^ (0.000)
Age	0.0007 (0.545)	0.0013 (0.236)	0.0043^*^ (0.097)	0.0002 (0.774)	0.0005 (0.545)	0.0001 (0.977)
Age^2^	0.0010 (0.393)	0.0009 (0.384)	0.0015^*^ (0.053)	0.0001 (0.879)	0.000 (0.380)	0.0046^*^ (0.068)
Gender	0.0221^***^ (0.003)	0.0108^***^ (0.113)	0.1013^***^ (0.000)	0.0153^***^ (0.005)	0.0103^**^ (0.032)	0.0822^***^ (0.000)
Y_E	−0.0005 (0.475)	−0.0010 (0.117)	− 0.0097^***^ (0.000)	− 0.0004 (0.504)	−0.0001 (0.248)	− 0.0105^***^ (0.000)
F_S	0.0209^***^ (0.000)	0.0176^***^ (0.000)	−0.0133^***^ (0.000)	0.0151^***^ (0.000)	0.0121^***^ (0.000)	−0.0438^***^ (0.000)
Loglikelihood	− 1282.257	− 1023.15	−3793.51	− 796.9304	− 611.08	− 3529.13
LR Statistics	329.57	1135.72	388.30	234.92	703.17	801.22
Number of Sampling	5754	5754	5754	5754	5754	5754

Source: CFPS 2014, 2016 and 2018 [56]

Note: *, **, *** represented statistic indicators significantly at 10, 5, and 1%, respectively

## Discussion

In this paper, our results show that there are obvious differences in the vulnerability of health care poverty among rural households in China. We estimate the vulnerability of rural households in China to multidimensional poverty and poverty in health care in 2014, 2016, and 2018 survey wave, respectively, and compared the vulnerability of 5754 rural households to health care poverty in 28 provinces and cities of the eastern, central and western regions during the 2018 survey wave. Regions such as Beijing and Shanghai, located in the eastern regions with high levels of economic development, have higher vulnerability to health care poverty, while western regions such as Xinjiang and Tibet, where economic development is low, have a lower vulnerability to health care poverty. This may be because, in rural areas with high poverty rates, such as Yunnan and Tibet, the level of economic development and population mobility are relatively low, with most families relying mainly on government financial assistance to support their survival and development, while family members have low opportunities to work outside the home for long periods. Then, family members have a lower risk of chronic and bronchitis illnesses. As a result, the incidence of rural family health care poverty vulnerability is low in these areas. Conversely, Beijing and Shanghai are the political and economic centers of China, respectively, and the two cities have high levels of economic development and high mobility [59]. Rural households in these areas receive less government financial assistance and are more likely to be employed in neighboring towns, which encourages rural family members to go out to work. In addition to these areas, high population concentration can easily lead to environmental pollution caused by smoking. Combined with these circumstances, it is easy to judge that rural households in these areas have a higher probability of chronic illness, bronchitis, and other illnesses, and may assess the high vulnerability of health care poverty in Beijing and Shanghai. This finding is consistent with the findings of [31]. Although Chaudhuri’s study measured the incidence of income poverty and the vulnerability of income poverty from an income poverty perspective, this paper measured the incidence of multidimensional poverty and the vulnerability of health care poverty in rural households. From Chaudhuri’s research, it has shown that areas with high incidences of poverty may experience lower levels of expected poverty vulnerability because of the direct correlation between the incidence of poverty and local levels of socio-economic development and government fiscal policies, while the expected vulnerability of poverty is based on the current study subjects’ situation, population mobility, etc.

From the return of the effect of public transfer payments on the health care vulnerability of rural households in China, it can be seen that government public transfer payments have had little effect on improving the vulnerability of rural family health care in 2014, 2016, and 2018 survey wave. This shows that the proportion of public transfer payments made by local governments to rural areas falls far short of the proportion of rural households that are expected to be vulnerable to health care. More importantly, it also explains the serious lack of public policy targeting by local governments, whose allocation of public transfers to rural households does not take into account the extent and differences in the needs of rural households for health care [60, 61]. Thus, it brings about the invalidation effect of public transfer payments. When compared to the previous studies, similar conclusions are drawn that predisposing factors are also the obvious factors the effect of public transfer payments on the vulnerability of health care to poverty. We show that the head of rural households for the younger and more educated female characteristics, government public transfer payments have been more effective than other situations in improving their vulnerability to health care poverty [62, 63]. However, our study applying income elasticity from rural households’ demand for health care into the multi-variables logistic regression model extended the findings. We find that rural households have greater income elasticity in their demand for health care, indicating that there may be a potential chronic risk or other potential health risks, resulting in a much greater demand for health care than they can afford to spend on it [64, 65]. Moreover, we show that the interaction between the elasticity of income elasticity of rural households’ demand for health care and the government’s public transfer payment has a significant effect on improving the vulnerability of rural households to health care poverty. It is suggesting that if the Government is aware of the differences in the demand for health care between different rural households, it should develop specific public mechanisms to target them. The effect of government public transfer payments on improving the vulnerability of rural households to health care poverty will then be efficient. These findings show novelty and difference in terms of the effects of public transfer payments on improving poverty vulnerability, in contrast to previous studies [31, 36, 61].

## Conclusions

In conclusion, we used data from the China Family Panel Studies (CFPS) in the 2016, 2014, and 2018 survey waves to prove the effect of government public transfer payments on the vulnerability of Chinese rural households to health care poverty. By estimating and comparing the incidence of health care multidimensional poverty and expected poverty vulnerability of rural households in China in three survey waves, it is found that the incidence of expected poverty vulnerability in the three study years is much higher than that of multidimensional poverty. Further, we classify the rural family health care poverty vulnerability in various provinces and cities in China. It is shown that the vulnerability of health care poverty is higher in areas with high economic development than in areas with low economic development. This situation may be closely related to the opportunities, intensity of work of members of rural households, public transfer payment support, and population mobility in the region.

We used two series of multi-variables logistics models to regress the marginal effects of public transfer payment to the vulnerability of rural family health care poverty. Series one of model results show that the government public transfer payment has little effect on improving the vulnerability of rural family health care poverty, the reason is that the public transfer payment input is less than the proportion of poverty vulnerability and the lack of public transfer payment targeting mechanism. Then through the series two models to make up for the imperfect public transfer payment targeting mechanism. Series two model includes the indicators of income elasticity of rural households’ health care demands and incorporates the income elasticity index of health care needs and the interaction between public transfer payments and their elasticity into the logistic model for regression analysis. The results show that the income elasticity of health care demand and the interaction between public transfer payments and their elasticity have significant effects on improving the vulnerability of rural households’ health care poverty.

This study has practical implications for policymakers and practitioners. Chinese government officials should optimize the identification mechanism of multidimensional poverty when implementing poverty alleviation measures for rural households. During the sample period, from 2014 to 2018, the average annual expenditure per unit of rural households on health care was RMB 5585, reflecting that rural households’ future demand payments on health may keep increasing, which may transform many potentially poverty households into multi-dimensionally poverty households deprived of health care [56]. Besides, local officials should effectively play the optimal implementation effect of public transfer policies. On the one hand, public transfer payments policy should not only bring financial incentive effect to local governments, but also guide the behavior of local government public sector and encourage them to increase the supply in health-related deprived rural households [66]; On the other hand, public transfer policy should focus on the difference in the degree of rural households’ demand for health care, and enhance the targeting of public transfer mechanism in the supply and demand of health care services. In this way, we can effectively improve the health care vulnerability poverty of rural households and improve people’s quality of life.

## Data Availability

The datasets used in this study were derived from China Family Panel Studies in the years 2014, 2016, and 2018. Available at: http:// www.isss.pku.edu.cn/cfps/. Accessed 10 Jan 2020. The code used in this study can be obtained from the corresponding author on reasonable request.

## Abbreviations

CFPS: China Family Panel Studies
ISSS: Institute of Social Science Survey
A-F: Alkire-Foster
MPI: Multidimensional Poverty Index
OPHI: Oxford Poverty & Human Development Initiative

## References

[1] “National Bureau of Statistics of China,” China Statistical Yearbook 2020. 2020. http://www.stats.gov.cn/tjsj/ndsj/2020/indexch.htm. Accessed 3 Jan 2021.

[2] Ravallion M, Jalan J (1996). Transient poverty in rural China. World Bank Policy Res Work Pap.

[3] Sen A (1976). Poverty: an ordinal approach to measurement. Econometrica.

[4] R. Warshaw, “Health Disparities Affect Millions in Rural U.S. Communities,” 2017. https://www.aamc.org/news-insights/health-disparities-affect-millions-rural-us-communities. Accessed 30 Mar 2021.

[5] Bird RM, Tarasov AV (2004). Closing the gap: fiscal imbalances and intergovernmental transfers in developed federations. Environ Plan C Gov Policy.

[6] Qian Y, Weingast BR (1997). Federalism as a commitment to reserving market incentives. J Econ Perspect.

[7] Dobriansky PJ, Suzman RM, Hodes RJ. Why Population Aging Matters - A Global Perspective. US Dep State. 2007:1–32 [Online]. Available: papers2://publication/uuid/4B8865DB-5866-4285-A74D-168F45ED1109.

[8] Wu I-C, Lin C-C, Hsiung CA (2015). Emerging roles of frailty and inflammaging in risk assessment of age-related chronic diseases in older adults: the intersection between aging biology and personalized medicine. BioMedicine.

[9] Jamison DT (2018). Disease Control Priorities. Improving health and reducing poverty.

[10] Sen A (1973). On economic inequality.

[11] United Nations DPI (2016). Transforming our world: 2030 agenda for sustainable development Department of Public Information United Nations.

[12] Alkire S, Santos ME (2014). Measuring acute poverty in the developing world: robustness and scope of the multidimensional poverty index. World Dev.

[13] Alkire S, Kanagaratnam U, Nogales R, Suppa N. Revising the global Multidimensional Poverty Index: Empirical insight and robustness, OPHI Res. Prog. 56a, Oxford Poverty Hum. Dev. Initiat. Univ. Oxford; 2020. p. 3. [Online]. Available: https://www.ophi.org.uk/wp-content/uploads/RP56a_Revising_2020.pdf. Accessed 10 Jan 2021.

[14] Alkire S, Kanagaratnam U (2020). Revisions of the global multidimensional poverty index: indicator options and their empirical assessment. Oxford Dev Stud.

[15] Alkire S, Kanagaratnam U, Suppa N (2020). The global Multidimensional Poverty Index (MPI): 2020 revision. *OPHI MPI Methodol. Notes 49, Oxford Poverty Hum. Dev*.

[16] Obi BO (2007). Fiscal Policy and Income Distribution: Some Policy Options for Nigeria.

[17] L. R. and T. S. Anthony Atkinson, “Income Distribution in Advanced Economies: The Evidence from the Luxembourg Income Study (LIS),” 1994.

[18] Dimova R, Wolff FC (2008). Are private transfers poverty and inequality reducing? Household level evidence from Bulgaria. J Comp Econ.

[19] Jung HS, Thorbecke E (2003). The impact of public education expenditure on human capital, growth, and poverty in Tanzania and Zambia: a general equilibrium approach. J Policy Model.

[20] Quisumbing AR (2003). Food aid and child nutrition in rural Ethiopia. World Dev.

[21] Parker D, Kirkpatrick C, Figueira-Theodorakopoulou C (2008). Infrastructure regulation and poverty reduction in developing countries: a review of the evidence and a research agenda. Q Rev Econ Financ.

[22] Jha R, Dang T, Tashrifov Y (2010). Economic vulnerability and poverty in Tajikistan. Econ Chang Restruct.

[23] Bronfman J. Measuring Vulnerability to Poverty in Chile Using the National Socio Economic Characterization Panel Survey for 1996, 2001, 2006. MPRA Paper No. 62689. 2015:2-20. https://mpra.ub.uni-muenchen.de/62689/.

[24] Grosh M, Hoddinott J, Ahmed A (2003). Targeting of transfers in developing countries : review of experience and lessons the targeting of transfers in developing Countries : review of experience and lessons David Coady Margaret Grosh John Hoddinott November 2003.

[25] Dabalen A, Kilic T, Wane W. Social Transfers, Labor Supply and Poverty Reduction; The Case of Albania, Policy Research Working Paper, 2008. [Online]. Available: https://documents1.worldbank.org/curated/en/246771468191366177/pdf/Social-transfers-labor-supply-and-poverty-reduction-the-case-of-Albania.pdf. Accessed 10 Sep 2020.

[26] Bargain O, Immervoll H, Viitamäki H (2012). No claim, no pain. Measuring the non-take-up of social assistance using register data. J Econ Inequal.

[27] Jonathan H, Khandker SR. Handbook on poverty and inequality. 2009. Washington, DC: World Bank. © World Bank. https://openknowledge.worldbank.org/handle/10986/11985.

[28] W. Naudé, A. U. Santos Paulino, and M. McGillivray, “MeasuringVulnerability : An Overview and Introduction,” 2014.

[29] Klasen S, Waibel H (2013). Vulnerability to poverty-theory, measurement and determinants, with case studies from Thailand and Vietnam.

[30] Ward PS (2016). Transient poverty, poverty dynamics, and vulnerability to poverty: an empirical analysis using a balanced panel from rural China. World Dev.

[31] Chaudhuri S, Jalan J, Suryahadi A. Assessing Household Vulnerability to Poverty from Cross-sectional Data: A Methodology and Estimates from Indonesia, *World*, 0102–52, April; 2002. p. 36. [Online]. Available: http://www.ncbi.nlm.nih.gov/entrez/query.fcgi?db=pubmed&cmd=Retrieve&dopt=AbstractPlus&list_uids=6086807498683160254related:vpYxtyuveFQJ. Accessed 08 Feb 2020.

[32] Ligon E, Schechter L (2003). Measuring vulnerability. Econ J.

[33] Grech A (2015). Evaluating the possible impact of pension reforms on elderly poverty in Europe. Soc Policy Adm.

[34] Pritchett L, Suryahadi A, Sumarto S. “Quantifying vulnerability to poverty: a proposed measure applied to Indonesia,” 2000. [Online]. Available: http://www.worldbank.org/research/workingpapers. Accessed 10 Oct 2020.

[35] Hoddinott J, Quisumbing A (2003). Data sources for microeconometric risk and vulnerability assessments.

[36] Chaudhuri S. Assessing vulnerability to poverty: concepts, empirical methods and illustrative examples. Dep Econ. 2003:56 [Online]. Available: http://info.worldbank.org/etools/docs/library/97185/keny_0304/ke_0304/vulnerability-assessment.pdf. Accessed 10 Sep 2020.

[37] Ravallion M, Chen S (2007). China’s (uneven) progress against poverty. J Dev Econ.

[38] Ziliak JP, Gundersen C, Smeeding T, Bartfeld J, Matters SNAP (2015). How food stamps affect health and well-being.

[39] Celidoni M (2013). Vulnerability to poverty: an empirical comparison of alternative measures. Appl Econ.

[40] Dercon S, Krishnan P (2000). Vulnerability, seasonality and poverty in Ethiopia. J Dev Stud.

[41] Bailey MJ, Danziger S, editors. Legacies of the war on poverty. New York: Russell Sage Foundation; 2013.

[42] Kakwani N, Subbarao K (2007). Poverty among the elderly in sub-Saharan Africa and the role of social pensions. J Dev Stud.

[43] Karim A, Noy I (2020). Risk, poverty or politics? The determinants of subnational public spending allocation for adaptive disaster risk reduction in Bangladesh. World Dev.

[44] Xiang Q, Yan C, Ma Y, Liao H, Wang J (2021). Classification and influencing factors of rural elderly’s vulnerability to health-related poverty in central and western regions of China. Glob Heal J.

[45] Harris H, Ooi YBH, Lee J-S, Matanjun P (2019). Non-communicable diseases among low income adults in rural coastal communities in eastern Sabah, Malaysia. BMC Public Health.

[46] Gloede O, Menkhof L, Waibel H (2012). Shocks, individual risk attitude, and vulnerability to poverty among rural households in Thailand and Vietna. Leibniz Univ Hann Discuss. Pap.

[47] Chen C, Pan J (2019). The effect of the health poverty alleviation project on financial risk protection for rural residents: evidence from Chishui City, China. Int J Equity Health.

[48] Zhang Y, Zheng X, Xie L (2021). How do poverty alleviation coordinators help the impoverished in rural China? -- Evidence from the Chinese poor population tracking dataset. China Econ Rev.

[49] Hashimoto K, Heath JA (1995). Income elasticities of educational expenditure by income class: the case of Japanese households. Econ Educ Rev.

[50] Christiaensen LJ, Subbarao K (2005). Towards an understanding of household vulnerability in rural Kenya. J Afr Econ.

[51] T. Amemiya, “The maximum likelihood and the nonlinear three-stage least squares estimator in the general nonlinear simultaneous equation model,” Econometrica, 45, 4, pp. 955–968, Jul. 1977, 10.2307/1912684.

[52] Andersen R (1968). A behavioral model of families’ use of health services.

[53] R. Andersen, “Revisiting the behavioral model and access to medical care: does it matter?,” J Health Soc Behav, 36, 1, pp. 1–10, Mar. 1995. 7738325

[54] Alkire S, Foster J (2011). Understandings and misunderstandings of multidimensional poverty measurement. J Econ Inequal.

[55] Oxford Poverty & Human Development Initiative, “Global Multidimensional Poverty Index 2020,”. 2020. https://www.ophi.org.uk/wp-content/uploads/OPHI_MPI_MN_49_2020.pdf. Accessed 25 Mar 2021.

[56] Institute of Social Science Survey (2021). China family panel studies.

[57] The World Bank, *piecing together the poverty puzzle*. 2018. https://openknowledge.worldbank.org/bitstream/handle/10986/30418/9781464813306.pdf. Accessed 10 Mar 2020.

[58] Lavrakas P (2008). Encyclopedia of survey research methods.

[59] Cao W, Feng X, Zhang H (2019). The structural and spatial properties of the high-speed railway network in China: a complex network perspective. J Rail Transp Plan Manag.

[60] Chen T, Wang Y, Luo X, Rao Y, Hua L (2018). Inter-provincial inequality of public health services in China: the perspective of local officials’ behavior. Int J Equity Health.

[61] Chen S, Li J, Lu S, Xiong B (2017). Escaping from poverty trap: a choice between government transfer payments and public services. Glob. Heal. Res. Policy.

[62] Dubikaytis T, Larivaara M, Kuznetsova O, Hemminki E (2010). Inequalities in health and health service utilisation among reproductive age women in St. Petersburg, Russia: a cross-sectional study. BMC Health Serv Res.

[63] Novignon J, Nonvignon J, Mussa R, Chiwaula LS (2012). Health and vulnerability to poverty in Ghana: evidence from the Ghana living standards survey round 5. Health Econ Rev.

[64] Lan X (2018). Assessing the effects of the percentage of chronic disease in households on health payment-induced poverty in Shaanxi Province, China. BMC Health Serv Res.

[65] Gong CH, Kendig H, He X (2016). Factors predicting health services use among older people in China: an analysis of the China health and retirement longitudinal study 2013. BMC Health Serv Res.

[66] Li T, Du T (2021). Vertical fiscal imbalance, transfer payments, and fiscal sustainability of local governments in China. Int Rev Econ Financ.

